# Adenoid Cystic Carcinoma of the Bartholin’s Gland: A Diagnostic Challenge

**DOI:** 10.7759/cureus.95975

**Published:** 2025-11-03

**Authors:** Hiroki Sato, Yuya Yamamoto, Kazuko Miyazaki, Mai Matsumura, Eito Kozawa

**Affiliations:** 1 Radiology, Saitama Medical University, Saitama, JPN; 2 Obstetrics and Gynecology, Saitama Medical University, Saitama, JPN; 3 Diagnostic Pathology, Saitama Medical University, Saitama, JPN

**Keywords:** adenoid cystic carcinoma, bartholin's gland, mri, vulvar mass, vulvar neoplasms

## Abstract

Adenoid cystic carcinoma (ACC) originating in the Bartholin’s gland is a rare entity. We report the case of a female in her 50s who presented with a progressively enlarging right-sided vulvar mass and intermittent pain. Although preoperative biopsy and MRI suggested a benign lesion, postoperative histopathological examination confirmed the diagnosis of ACC. ACC can be challenging to diagnose using MRI, and misidentifying it as a benign lesion can lead to insufficient surgical treatment and a poorer prognosis. Therefore, ACC should be included in the differential diagnosis of vulvar soft-tissue masses when the lesion shows slow, progressive enlargement.

## Introduction

Adenoid cystic carcinoma (ACC) is a malignant tumor that most frequently originates in the salivary glands but may also occur in the paranasal sinuses, lacrimal glands, and tracheobronchial tree. [1]. In contrast, Bartholin’s gland ACC (BG-ACC) is extremely rare [2]. Clinically, the initial manifestations of BG-ACC often resemble those of benign Bartholin’s gland cysts or abscesses, and imaging findings are commonly nonspecific, leading to diagnostic delays or misclassification as a benign lesion [2]. We report a case of BG-ACC that was initially misinterpreted as a benign lesion on preoperative biopsy and MRI. Reports on the imaging findings of this disease are extremely scarce, and we discuss our case in the context of existing literature.

## Case presentation

A female in her 50s (gravida 2, para 1) with no significant medical history other than a prior hysteroscopic myomectomy first noticed a right vulvar mass approximately two years earlier. At the referring hospital, pelvic examination and contrast-enhanced MRI revealed a 40-mm subcutaneous mass in the right perineum. Biopsy revealed loose connective tissue with no evidence of malignancy, and the patient was managed conservatively with observation. Over time, the mass gradually increased in size, and intermittent local pain developed, prompting referral to our institution.

Upon presentation, visual inspection revealed no visible mucosal abnormalities. Palpation revealed a firm mass with an irregular surface that extended from the medial aspect of the right labium to the adjacent vaginal wall. Results of routine blood tests, including complete blood count, biochemistry, and coagulation parameters, were unremarkable; serum tumor markers were not assessed. Transperineal ultrasonography (images not archived) revealed a well-circumscribed, slightly hypoechoic mass with internal blood flow in the subcutaneous tissue of the right perineum.

**Figure 1:**
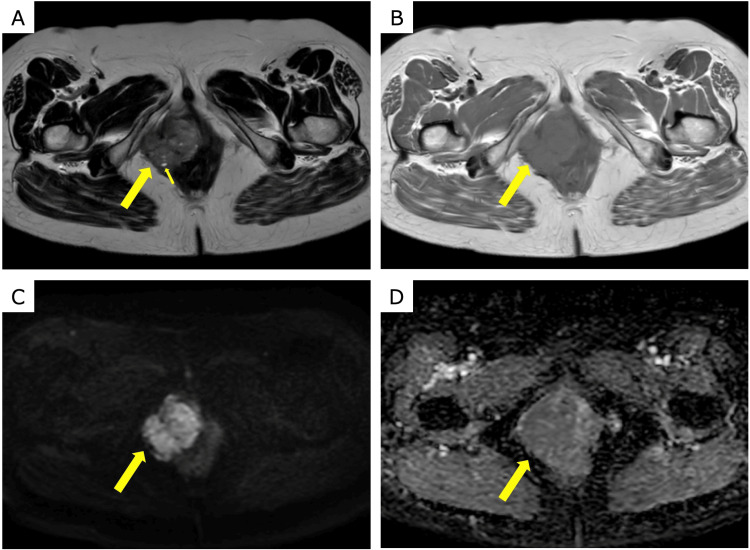
Pelvic MRI performed at our institution (A) T2-weighted image showing heterogeneous signal intensity, predominantly mildly hypointense with areas of mild hyperintensity, including small high-signal foci (small arrow) suggestive of cystic components. (B) Unenhanced T1-weighted image showing that the lesion is isointense to the adjacent muscle, with no fat or hemorrhagic components. (C, D) Diffusion-weighted image (C, b = 1000 s/mm²) shows high signal, whereas the apparent diffusion coefficient map (D) shows a value of 1.23 × 10⁻³ mm²/s, without restricted diffusion MRI: magnetic resonance imaging

Contrast-enhanced MRI at our institution showed mild interval growth (maximum diameter: 50 mm). On T2-weighted imaging (T2WI), the mass showed heterogeneous signal intensity, was mainly mildly hypointense with some areas of hyperintensity, and included small high-signal foci, suggesting cystic components (Figure 1A). On T1-weighted imaging (T1WI), the lesion was isointense with the adjacent muscle and had no fat or hemorrhagic components (Figure 1B). The lesion was hyperintense on diffusion-weighted imaging (DWI); the apparent diffusion coefficient (ADC) measured 1.23 × 10^-3 ^mm^2^/s, with no evidence of restricted diffusion (Figures 1C, 1D).

**Figure 2:**
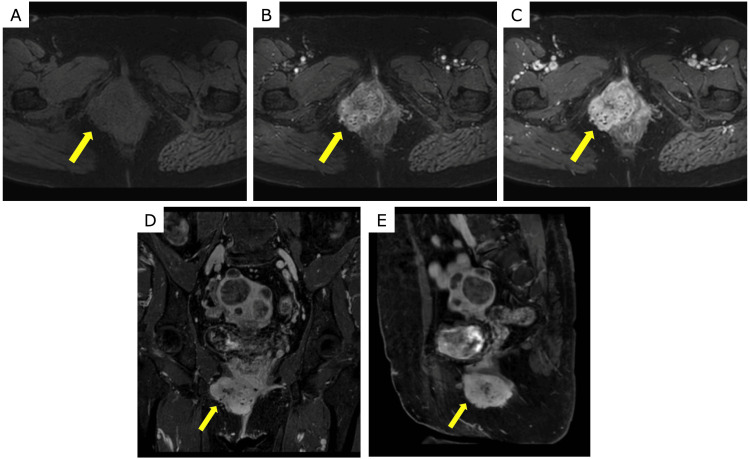
Dynamic contrast-enhanced and post-contrast images (A-C) Fat-suppressed dynamic contrast-enhanced T1-weighted images (A, pre-contrast; B, 30 s post-injection; C, 120 s) show a gradual enhancement pattern. (D, E) Fat-suppressed post-contrast T1-weighted images in the coronal (D) and sagittal (E) planes show that the mass has lobulated margins and abuts the right vaginal wall, without definite invasion of the bladder, rectum, or pelvic floor musculature

The dynamic sequences showed a gradual enhancement pattern (Figures 2A, 2B, 2C). The lesion had lobulated margins and abutted the right vaginal wall. However, there was no definite invasion of the bladder, rectum, or pelvic floor musculature (Figures 2D, 2E). No significant pelvic lymphadenopathy was observed in the patient. The primary preoperative differential diagnoses included benign tumors such as angiomyofibroblastoma, cellular angiofibroma, and aggressive angiomyxoma. In general, for suspected vulvar malignancy, preoperative staging CT is often considered; however, because a benign lesion was suspected in this case, preoperative CT was not performed. Local excision of the tumor was performed for diagnostic and therapeutic purposes.

**Figure 3:**
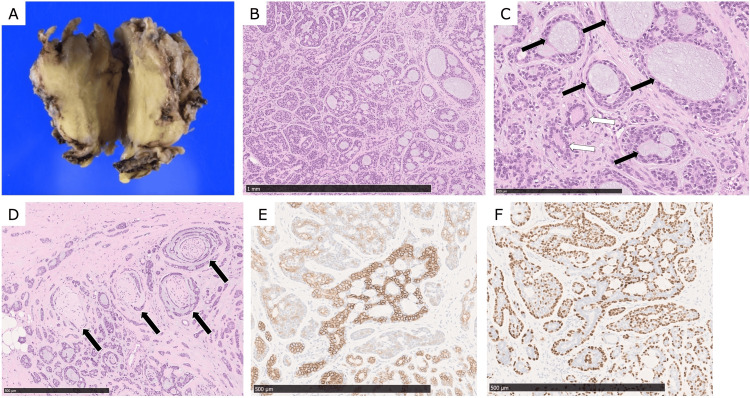
Histopathology and immunohistochemistry (A) The resected specimen measured 56 × 46 × 35 mm, and the cut surface had a whitish, solid-cut appearance. (B, C) Hematoxylin and eosin (H&E) staining shows a cribriform pattern composed predominantly of myoepithelial cells with myxoid globules (black arrows). Tubular patterns composed of inner ductal and outer myoepithelial cells are also present (white arrows). (D) H&E staining shows marked perineural invasion (black arrows). (E) Immunohistochemistry shows c-KIT (CD117) positivity in ductal epithelial cells. (F) Immunohistochemistry shows p63 positivity in the myoepithelial cells surrounding the pseudolumen of cribriform patterns and in the outer myoepithelial cells of tubular patterns

The resected mass measured 56 × 46 × 35 mm and had a whitish, solid-cut surface (Figure 3A). Histologically, the tumor was a biphasic salivary gland tumor composed of ductal and myoepithelial cells forming cribriform, tubular, and cord-like structures with infiltrative growth (Figures 3B, 3C). Extensive perineural and vascular invasions were observed (Figure 3D). Based on these characteristic histologic features, ACC was strongly suspected. Immunohistochemically, the ductal cells were positive for c-KIT (CD117) and epithelial membrane antigen (EMA), whereas the myoepithelial cells were positive for p63, α-smooth muscle actin (α-SMA), calponin, and glial fibrillary acidic protein (GFAP) (Figures 3E, 3F). These findings confirmed a diagnosis of ACC. The surgical margins were initially reported as negative at our institution.

The patient was subsequently referred to a specialized hospital for further management. At that hospital, a follow-up MRI two months after surgery demonstrated a local recurrence adjacent to the surgical site. Reevaluation of the pathological specimen suggested a positive margin. Therefore, due to the potential presence of residual disease, additional radiotherapy was administered.

## Discussion

Vulvar cancers account for approximately 4% of all gynecological malignancies [3], and BG-ACC constitutes only 0.1%-5% of vulvar cancers [2], underscoring its rarity. The median age of BG-ACC patients is 48 years (range: 25-80 years), and the age distribution does not show a particular pattern [4]. Early BG-ACC often presents as a Bartholin’s gland cyst or abscess; consequently, it is frequently misdiagnosed as a benign lesion [2]. Several reports have described BG-ACC cases diagnosed as benign before surgery, similar to our patient [2,5,6]. Furthermore, approximately 33% of salivary gland ACC cases have reportedly been misdiagnosed as benign on fine-needle aspiration [7], suggesting that ACC may be difficult to diagnose, even on biopsy.

ACC typically demonstrates slow growth compared to other carcinomas and has a relatively low rate of lymph node metastasis. Nonetheless, both local and distant recurrences are frequently observed after primary tumor resection. This high recurrence rate is thought to reflect the tumor’s tendency for perineural or perivascular invasion with occult extension beyond the surgical margins [1]. Symptoms associated with perineural invasion, including local itching and burning sensations, have been reported [5], and the intermittent pain experienced by our patient may have been a manifestation of this process.

The MRI features of BG-ACC have not been well characterized, owing to the limited number of reported cases. At other anatomic sites, the ACC typically appears iso- to hypointense on T1-weighted images, whereas the T2-weighted signal intensity is variable, with many cases demonstrating T2 hyperintensity [8,9]. ACC often shows high ADC values without diffusion restriction, potentially mimicking benign lesions; this imaging appearance is thought to reflect the presence of cystic changes or myxoid stroma [10,11]. Dynamic enhancement patterns commonly include gradual or early enhancement with low washout [12,13]. As mentioned above, ACC shows a propensity for perineural invasion [1], and BG-ACC with MRI evidence of perineural spread has been described [14].

In the present case, T1 isointensity, a relatively high ADC value, and a gradual enhancement pattern were consistent with reported ACC features. In contrast, no linear enhancement along the neural pathways was observed to suggest perineural spread, and no invasion of adjacent pelvic organs or the pelvic floor was identified, despite the tumor abutting the right vaginal wall. The imaging differential diagnoses for vulvar soft tissue masses, such as in the present case, include aggressive angiomyxoma (AA), angiomyofibroblastoma (AMF), and cellular angiofibroma (CAF). AA often demonstrates an internally layered or swirling architecture on T2-weighted and contrast-enhanced T1-weighted images, a feature reported in approximately 83% of cases that reflects its fibrous and vascular stroma [15]. AMF and CAF are usually well circumscribed with limited local infiltration [16,17]. CAFs are typically small, with a mean diameter of approximately 3 cm [17], and may exhibit characteristic tortuous, peritumoral vessels [18]; however, this finding is not universal.

Surgical resection is the mainstay of BG-ACC treatment [2], and adjuvant radiotherapy is recommended in cases of positive surgical margins or perineural invasion [5]. Surgical approaches are usually divided into local excision and radical vulvectomy; compared with local excision, radical vulvectomy has been associated with lower rates of recurrence and positive surgical margins. In one study, local excision was associated with a recurrence rate of 69.8% and a positive margin rate of 48%, whereas radical vulvectomy was associated with lower rates of 42.9% and 30%, respectively [2]. Furthermore, it has been reported that the median survival was 31 years in patients with negative margins, whereas it was eight years in those with positive margins, indicating a marked difference in prognosis [4]. Therefore, if serial clinical or imaging evaluations demonstrate gradual progressive enlargement, ACC should be considered in the preoperative differential diagnosis to inform appropriate surgical management.

## Conclusions

This report illustrates how BG-ACC can closely mimic benign vulvar soft tissue tumors on MRI, making preoperative diagnosis challenging. Given that ACC may also be misinterpreted as benign on biopsy, this diagnostic difficulty may lead to suboptimal surgical management and adversely affect recurrence risk and long-term prognosis. Hence, when a vulvar mass exhibits slow, progressive enlargement, ACC should be considered in the differential diagnosis.

## Appendices

Technical information about the MRI

Pelvic MRI was performed at 3T (Philips Ingenia Elition X) using a receive coil (“MULTI COIL”). Sequences and key parameters were as follows: T1WI (TR 1658.8 ms, TE 10 ms, flip angle 90°, slice thickness 5 mm, intersection gap 7 mm); T2WI (TR 5940.8 ms, TE 100 ms, flip angle 90°, slice thickness 5 mm, intersection gap 7 mm); DWI (TR 6750 ms, TE 75 ms, flip angle 90°, slice thickness 5 mm, intersection gap 7 mm; b = 1000 s/mm²), with ADC maps generated from b = 0/1000 s/mm²; dynamic contrast-enhanced T1WI at 0 s, 30 s, and 120 s (TR 4.1253 ms, TE 1.875 ms, flip angle 10°, slice thickness 1.5 mm, intersection gap 1 mm for each time point); and post-contrast T1WI in the coronal plane (TR 3.9968 ms, TE 1.7447 ms, flip angle 12°, slice thickness 0.9 mm, intersection gap 0.9 mm) and sagittal plane (TR 3.9968 ms, TE 1.7447 ms, flip angle 12°, slice thickness 3 mm, intersection gap 3 mm).
